# Advances in Oral Drug Delivery for Regional Targeting in the Gastrointestinal Tract - Influence of Physiological, Pathophysiological and Pharmaceutical Factors

**DOI:** 10.3389/fphar.2020.00524

**Published:** 2020-04-28

**Authors:** Susan Hua

**Affiliations:** ^1^Therapeutic Targeting Research Group, School of Biomedical Sciences and Pharmacy, University of Newcastle, Callaghan, NSW, Australia; ^2^Hunter Medical Research Institute, New Lambton Heights, NSW, Australia

**Keywords:** gastrointestinal, oral, drug delivery, gastroretentive, small intestine, colon, nanomedicine, formulation, translation

## Abstract

The oral route is by far the most common route of drug administration in the gastrointestinal tract and can be used for both systemic drug delivery and for treating local gastrointestinal diseases. It is the most preferred route by patients, due to its advantages, such as ease of use, non-invasiveness, and convenience for self-administration. Formulations can also be designed to enhance drug delivery to specific regions in the upper or lower gastrointestinal tract. Despite the clear advantages offered by the oral route, drug delivery can be challenging as the human gastrointestinal tract is complex and displays a number of physiological barriers that affect drug delivery. Among these challenges are poor drug stability, poor drug solubility, and low drug permeability across the mucosal barriers. Attempts to overcome these issues have focused on improved understanding of the physiology of the gastrointestinal tract in both healthy and diseased states. Innovative pharmaceutical approaches have also been explored to improve regional drug targeting in the gastrointestinal tract, including nanoparticulate formulations. This review will discuss the physiological, pathophysiological, and pharmaceutical considerations influencing drug delivery for the oral route of administration, as well as the conventional and novel drug delivery approaches. The translational challenges and development aspects of novel formulations will also be addressed.

## Introduction

The oral route is by far the most common route for drug administration in the gastrointestinal tract (GI tract) and can be used for both systemic drug delivery and for treating local gastrointestinal diseases. It is the most preferred route by patients, due to its advantages, such as ease of use, non-invasiveness, and convenience for self-administration (Shreya et al., 2018; Homayun et al., 2019). Formulations can also be designed to enhance drug delivery to specific regions in the upper or lower GI tract. The upper GI tract consists of the mouth, pharynx, esophagus, stomach, and the first part of the small intestine (duodenum), whereas the lower GI tract includes the other parts of the small intestine (jejunum and ileum) and the large intestine (cecum, colon, and rectum) (Marieb and Hoehn, 2010; Reinus and Simon, 2014). Drugs administered *via* the oral route, however, generally have slower absorption, which is not preferred during an emergency (Homayun et al., 2019). They might also be unpleasant in taste, cause gastric irritation, and/or undergo first-pass drug elimination processes in both the intestine and liver (Martinez and Amidon, 2002; Homayun et al., 2019). In addition, the physiological environment in the GI tract can also affect the stability and solubility of drugs (Martinez and Amidon, 2002; Shreya et al., 2018; Homayun et al., 2019).

There are generally three main goals in formulation design for the oral route of gastrointestinal drug delivery (Martinez and Amidon, 2002): (i) local drug delivery to treat gastrointestinal disease, whereby the drug generally needs to be taken up into gastrointestinal mucosa but will not be systemically absorbed or will be poorly absorbed; (ii) systemic drug delivery, where drug absorption needs to be able to traverse the mucosal wall into the systemic circulation; and (iii) increase dissolution rate of poorly soluble drugs, which generally does not require the formulation to cross the mucosa or cells. Drug absorption in the GI tract is governed by many factors such as surface area for absorption, blood flow to the site of absorption, the physical state of the drug (such as a solution, suspension or solid dosage form), its water solubility, and the concentration of the drug at the site of absorption (Martinez and Amidon, 2002; Brunton et al., 2018). For absorption to occur, drugs must be able to penetrate the epithelium, which is the innermost layer that forms a continuous lining of the entire GI tract. This epithelial cell barrier selectively regulates transport from the lumen to the underlying tissue compartment. Drug molecules can be transported passively *via* paracellular diffusion (between cells) and transcellular diffusion (through the cell) or actively *via* receptor-mediated endocytosis and carrier-mediated transport. Of these pathways, the transcellular route is the main mechanism of drug absorption in the GI tract and is usually proportional to the lipid solubility of the drug (Brunton et al., 2018; Homayun et al., 2019). Therefore, absorption is favored when the drug molecule is in the non-ionized form, which is much more lipophilic than the ionized form.

Oral drug delivery is a significant area of formulation research due to the aforementioned advantages for patients. Significant pharmaceutical advances have been made to improve the regional targeting of drugs in the GI tract, however very few of them have translated to the clinical phase. This review will discuss the physiological, pathophysiological, and pharmaceutical considerations influencing drug delivery for the oral route of administration, as well as the conventional and novel drug delivery approaches. The translational challenges and development aspects of novel formulations will also be addressed.

## Functional Anatomy

The GI tract is a muscular tube that is approximately 9 meters in length with varying diameters. The main functions of the GI tract are the digestion of food, absorption of nutrients, and excretion of waste products (Marieb and Hoehn, 2010; Reinus and Simon, 2014). Following oral administration, food and pharmaceuticals transit through the esophagus to the stomach, aided by peristaltic contractions. Most of the digestion then takes place in the stomach by the action of acid and enzymes, especially peptidases (Reinus and Simon, 2014). The stomach also acts as a temporary reservoir for ingested food before it is delivered to the duodenum at a controlled rate. Very little drug absorption occurs in the stomach owing to its small surface area.

The small intestine is the longest (approximately 6 meters in length) and most convoluted part of the GI tract, where digestion is completed with enzymes from the liver and the pancreas, and most of the absorption of nutrients then takes place (Marieb and Hoehn, 2010; Reinus and Simon, 2014). The small intestine is also the major site of drug absorption, due to its large surface area. The surface area of the small intestine is increased enormously to approximately 200 m^2^ in an adult owing to the presence of villi and microvilli that are well supplied with blood vessels (Marieb and Hoehn, 2010; Reinus and Simon, 2014). Villi are finger-like projections that protrude into the intestinal lumen and are covered by epithelial cells. Interestingly, Helander et al. recently recalculated the mucosal surface area of the intestine in humans using morphometric data obtained by light and electron microscopy on biopsies from healthy adult volunteers or patients with endoscopically normal mucosae. They reported a mean total mucosal surface area of approximately 32 m^2^ for the interior of the GI tract, with approximately 2 m^2^ representing the large intestine (Helander and Fandriks, 2014).

The large intestine is the final major part of the GI tract. Its primary function is to process the waste products and absorb any remaining nutrients and water back into the system, which is important for homeostasis (Reinus and Simon, 2014). The remaining waste is then sent to the rectum and discharged from the body as stool. The colon has been investigated as a site for both systemic and local drug delivery. Anatomically, it can be further divided into four parts — ascending, transverse, descending, and sigmoid colon. The mucosa of the colon is smooth and has no specialized villi, hence the surface area is vastly smaller than the small intestine (Marieb and Hoehn, 2010; Reinus and Simon, 2014). However, the surface area of the large intestinal epithelium is amplified by being arranged into crypt structures. The colon is permanently colonized by an extensive number and variety of bacteria, which form the microbiome (Consortium, 2012; Reinus and Simon, 2014).

## Physiological Factors Influencing Oral Drug Delivery

**Figure 1:**
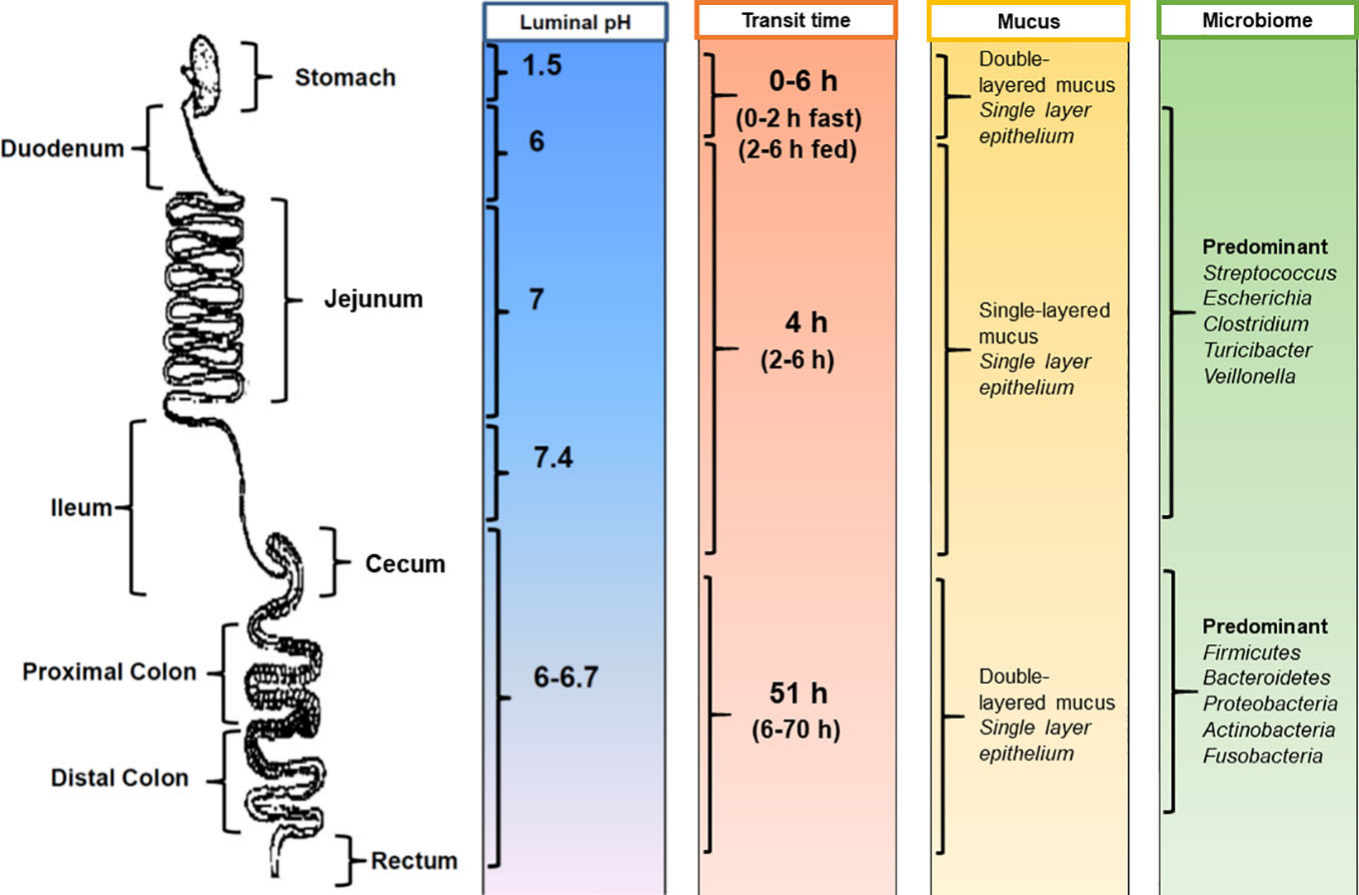
Physiological factors in the gastrointestinal tract that influence oral drug delivery. [Adapted from (Hua et al., 2015)].

Despite the clear advantages offered by the oral route, drug delivery can be challenging as the human GI tract is complex and displays a number of physiological barriers that affect drug delivery. Among these challenges are poor drug solubility, poor drug stability, and low drug permeability across the mucosal barriers (Martinez and Amidon, 2002). Even within healthy individuals, there is variability in the physiology of the GI tract (**Figure 1**). Therefore, considerations should be made during formulation design to the following factors (Martinez and Amidon, 2002; Hua et al., 2015): (i) how long the formulation resides in specific sections of the GI tract; (ii) the influence of the gastrointestinal environment on the delivery of the formulation at the site of action as well as on the stability and solubility of the drug; (iii) the intestinal fluid volume; and (iv) the degree of metabolism of the drug or formulation in the GI tract through microbial or enzymatic degradation.

### Gastrointestinal Transit Time

Gastrointestinal transit time is an important factor for dosage forms and drugs that have region-specific targeting or absorption properties (**Figure 1**). The amount of time needed for a dosage form to leave the stomach is highly variable and can range from several minutes to several hours (Reinus and Simon, 2014). Gastric transit time depends on many physiological factors, including age, body posture, gender, osmolarity, and food intake (Timmermans and Moes, 1994; Kagan and Hoffman, 2008). For example, gastric transit can range from 0 to 2 h in the fasted state and can be prolonged up to 6 h in the fed state (Reinus and Simon, 2014). In general, the transit time in the small intestine is considered relatively constant at around 3 to 4 h (Hu et al., 2000). However, this can range from 2 to 6 h in healthy individuals (Reinus and Simon, 2014). Colonic transit times can be highly variable, with ranges from 6 to 70 h reported (Coupe et al., 1991; Rao et al., 2004). Additional confounders affecting gastrointestinal transit time include the time of dosing in relation to an individual's bowel movements (Sathyan et al., 2000) and gender, with females having significantly longer colonic transit times (Buhmann et al., 2007).

### Gastrointestinal pH

With regards to the gastrointestinal environment, differences in pH along the GI tract have been exploited for the purposes of delayed release therapies (**Figure 1**). The highly acidic gastric environment (pH 1.5–2 in the fasted state) rises rapidly to pH 6 in the duodenum and increases along the small intestine to pH 7.4 at the terminal ileum (Fallingborg et al., 1993; Bratten and Jones, 2006). It should be noted that the pH in the cecum drops just below pH 6 and again rises in the colon reaching pH 6.7 at the rectum (Evans et al., 1988; Sasaki et al., 1997; Nugent et al., 2001). However, individuals can exhibit variability in pH ranges, with factors such as dietary intake (i.e., food and fluids) as well as microbial metabolism being major determinants (Ibekwe et al., 2008). For example, gastric pH can increase to 3–6 in the fed state. Gastrointestinal pH can also affect the ionization state of drug molecules, which in turn influences drug absorption (Brunton et al., 2018).

### Gastrointestinal Mucus

The continuous secretion of mucus in the GI tract is another hurdle for the effective oral delivery of drugs. Mucus secretion acts as a lubricant to facilitate the passage of digestive matter and to protect the underlying epithelium from pathogens and mechanical stress (Atuma et al., 2001). The mucus is composed of water and mucin protein molecules coated with proteoglycans, which gives the mucus a negative charge (Homayun et al., 2019). The mouth and esophagus do not have a distinct mucus layer, but they are washed by mucus from the salivary glands (Johansson et al., 2013). The small intestine has only one type of mucus that is unattached and loose (Atuma et al., 2001; Johansson et al., 2013). In contrast, the stomach and colon have the thickest mucus layer in the GI tract, with a two-layered mucus system comprising of: (i) an inner, attached mucus layer and (ii) an outer, unattached, loose mucus layer (Atuma et al., 2001; Johansson et al., 2013) (**Figure 1**). The thick mucus layer protects the mucosal tissue from gastric acid in the stomach and also provides a stable environment for the enteric microflora in the colon (Macfarlane et al., 1988; Atuma et al., 2001). It should be noted that mucus is continuously secreted by goblet cells along the GI tract and is subsequently shed and cleared from tissues due to the turnover of cells (Homayun et al., 2019). For drug delivery, the mucus layer acts as an important barrier for the permeability of drug molecules (especially hydrophobic molecules) and can also decrease the residence time of drugs and dosage forms.

### Intestinal Fluid Volume

Control of luminal fluidity is central to gastrointestinal function (Chowdhury and Lobo, 2011; Reinus and Simon, 2014). For example, the fluid environment permits contact of digestive enzymes with food particles, assists in the transit of intestinal contents along the length of the GI tract without damage to the epithelial lining, and supports the dissolution and absorption of nutrients and drugs (Reinus and Simon, 2014). Daily water balance in the healthy adult human GI tract includes secretion from saliva (1.5 L), gastric juice (2.5 L), pancreatic juice (1.5 L) and other intestinal components (~1 L), as well as absorption from the small intestine (7 L) and large intestine (1.9 L) (Reinus and Simon, 2014). Fluid-to-matter ratios influence pH and may also affect drug delivery and drug absorption, particularly in the lower GI tract. For example, food intake can significantly alter free fluid volumes, bile salts, and digestive enzyme levels in the GI tract (Reinus and Simon, 2014). In addition, the viscosity of the mucous-gel layer is affected by intestinal fluid secretion (Johansson et al., 2013; Reinus and Simon, 2014), which may influence the ability of drugs to be taken up by cells at the site of action. Increased fluid secretion and decreased reabsorption can dilute digestive enzymes and alter the intestinal microbiome. This can affect carbohydrate and polysaccharide digestion (Yang, 2008) as well as contribute to changes in intestinal transit times (Van Citters and Lin, 2006). Therefore, changes in intestinal fluid volumes can influence the way conventional formulations are processed in the GI tract.

### Gastrointestinal Enzymes and Microbiome

Enzymatic and microbial degradation of drugs and dosage forms can occur throughout the GI tract. **Table 1** shows the main enzymes in the saliva, gastric fluid, and intestinal fluid that are important in the metabolism of proteins, fats, and carbohydrates. The stomach and small intestine are the site of action for the major enzymes involved in the digestion of food (Marieb and Hoehn, 2010; Reinus and Simon, 2014). These enzymes can affect the stability of susceptible drugs and dosage forms, but they can also be exploited in formulation design for regional drug delivery in the GI tract.

**Table 1:** Main enzymes in the gastrointestinal tract.

Enzyme	Produced by	Site of action
Salivary amylase	Salivary glands	Mouth
Pancreatic amylase	Pancreas	Small intestine
Maltase	Small intestine	Small intestine
Pepsin	Gastric glands	Stomach
Trypsin	Pancreas	Small intestine
Peptidases	Small intestine	Small intestine
Nuclease	Pancreas	Small intestine
Nucleosidases	Small intestine	Small intestine
Lipase	Pancreas	Small intestine

The intestinal microbiome, which contains over 500 distinct bacterial species (Sartor, 2008; Consortium, 2012), is also important for both digestion and intestinal health, including digestion and metabolism of carbohydrates, fatty acids, and proteins (Macfarlane and Macfarlane, 2011) (**Figure 1**). The majority of the intestinal microbiome resides in the anaerobic colon and fermentation of carbohydrates is the main source of nutrition for this population (Macfarlane and Macfarlane, 2011). This has been exploited in formulation design with the use of non-starch polysaccharide coatings, which undergo relatively exclusive fermentation by the colonic microbiome (Sinha and Kumria, 2001). Both genetic and environmental factors contribute to the considerable variation in the composition of the microbiome that is seen between individuals (Sartor, 2010). However, the dominant species (*Firmicutes*, *Bacteroidetes*, *Proteobacteria*, *Actinobacteria*, and *Fusobacteria*) appear to be consistent and represent the majority of the colonic flora (Frank et al., 2007; Consortium, 2012).

Interestingly, the gastrointestinal microbiome not only resides in the large intestine but is also found in the small intestine. In comparison to the large intestine, the density of the small intestinal microbiota is much lower, which is likely due to the rapid luminal flow, intestinal fluid volume, and the secretion of bactericidal compounds in this part of the GI tract (El Aidy et al., 2015). In addition, the composition of the microbiome in the small intestine can significantly fluctuate over a short period of time (e.g., within a day to several days) and is influenced by variations in dietary intake (Booijink et al., 2010). The small intestinal microbiota (El Aidy et al., 2015) is predominantly composed of subject-specific genera such as Clostridium, Escherichia, and Turicibacter in variable amounts. Streptococcus and Veillonella species are also consistently found in the small intestine. This endogenous microenvironment is thought to play a pivotal role in metabolic regulation (El Aidy et al., 2015). The effect of the small intestinal microbiota on oral dosage forms and drug absorption has not yet been elucidated.

## Pathophysiological Factors Influencing Oral Drug Delivery

Adding to this complexity are the changes in gastrointestinal physiology associated with gastrointestinal or systemic disease, concurrent medications, and gastrointestinal surgery. These factors are dynamic, inter-related, and can further affect the efficacy of orally administered formulations. Therefore, they remain an important challenge in formulation design.

### Impact of Disease on Oral Drug Delivery

Depending on disease severity, gastrointestinal pathologies can affect some or all of the physiological variables for oral drug delivery (Hatton et al., 2018). For example, many acute gastrointestinal infections can cause temporal impairment in the microbiome (dysbiosis) (Britton and Young, 2014), drive increased intestinal fluid secretion (Patel and McCormick, 2014), and may increase or decrease bowel motility (Grover et al., 2008; Albenberg and Wu, 2014). These can affect the performance of locally acting dosage forms. For example, increased colonic motility in diarrhea can lead to reduced retention of locally acting dosage forms and incomplete drug release (Watts et al., 1992). In addition, toxins secreted by intestinal pathogens can cause intestinal inflammation and increased epithelial permeability, which may alter the concentration of drugs in the colonic mucosa (Hoque et al., 2012; Hatton et al., 2018).

In contrast, chronic diseases, such as inflammatory bowel disease (IBD), can cause significant changes to the physiology of the GI tract. IBD encompasses a group of chronic relapsing gastrointestinal diseases. The two main subtypes of IBD are Crohn's disease and ulcerative colitis (Podolsky, 2002). Both are considered distinct conditions, however, they can display many similar clinical features and typically result in cycles of remitting and relapsing inflammation of the mucosal tissue. The inflammation is continuous in UC and is confined to the colon (Podolsky, 2002). In some cases, the entire colon can also be affected (pancolitis). Crohn's inflammation, however, is generally discontinuous in manner and can affect any region of the GI tract. The commonly affected regions include the terminal ileum and the colon (Podolsky, 2002). The physiological changes associated with chronic inflammation of the GI tract should be considered in the development of improved oral delivery strategies for the management of IBD (Hua et al., 2015). Mucosal inflammation in IBD causes pathophysiological changes, such as: (i) increased mucus production; (ii) a disrupted intestinal barrier due to the presence of mucosal surface alterations, ulcers, and crypt distortions; and (iii) infiltration of immune cells (e.g., macrophages, lymphocytes, neutrophils, and dendritic cells) (Li and Thompson, 2003; Antoni et al., 2014). Together these changes can increase colonic epithelial permeability.

During relapse of IBD, patients suffering from severe mucosal inflammation may exhibit altered gastrointestinal motility and diarrhea, which in turn affects intestinal volume, pH, and mucosal integrity (Hua et al., 2015). In general, delayed orocecal transit times (i.e., the time taken for the meal to reach the cecum) have been reported in IBD patients, except when patients experience dysbiotic conditions (e.g., small intestinal bacterial overgrowth, SIBO) which can be associated with faster transit times (Kashyap et al., 2013; Rana et al., 2013). Studies have also shown that the colonic pH in IBD patients can be highly variable in terms of disease progression and severity, with some patients having more acidic colonic pH in the range of 2.3–5.5 (Fallingborg et al., 1993; Sasaki et al., 1997; Nugent et al., 2001). The inflammatory response at the mucosa, along with severe diarrhea, will also disrupt the resident microbiome by affecting the composition and diversity of the bacterial species (Linskens et al., 2001). This, in turn, can alter microbial metabolism in the GI tract and affect the secretion of enzymes. Therefore, active inflammation significantly alters the physiology of the GI tract, which can particularly affect the efficacy of conventional oral drug delivery approaches (Hua et al., 2015).

Furthermore, there is increasing evidence showing that non-gastrointestinal systemic diseases can also cause physiological and functional changes in the GI tract that can affect the performance of oral dosage forms and the absorption of drugs. This includes cystic fibrosis, Parkinson's disease, diabetes, HIV infection, and pain (Hatton et al., 2019). For example, pain can alter gastrointestinal physiology by affecting motility, secretion, intestinal permeability, mucosal blood flow, and the intestinal microbiome (Konturek et al., 2011).

### Impact of Drugs on Oral Drug Delivery

Drugs can alter the physiology of the GI tract and affect the performance of other co-administered oral dosage forms and the absorption of other drugs. For example, drugs used to reduce gastric acid secretion (e.g., proton pump inhibitors and histamine H2-receptor antagonists) or modify pH (e.g., antacids) (Lahner et al., 2009; Brunton et al., 2018) can affect dosage forms that rely on the difference in pH in various regions of the GI tract to trigger drug release. Drugs that alter the motility of the GI tract can also have an impact on the effectiveness of oral drug delivery by affecting the time available for disintegration, dissolution, and/or drug absorption (Watts et al., 1992; Brunton et al., 2018). This includes the following: (i) drugs that act as prokinetics to stimulate gastrointestinal motility (e.g., metoclopramide, domperidone, and cisapride); (ii) drugs that can cause constipation (e.g., opioids, anticholinergic agents, antidiarrheal agents, antacids containing aluminium or calcium, iron/calcium supplements, diuretics, verapamil, and clonidine); and drugs that can cause diarrhea (e.g., laxatives, antibiotics, colchicine, cytotoxic agents, digoxin, magnesium, NSAIDs, orlistat, acarbose, and metformin). In addition, administration of antibiotics can cause dysbiosis (Sartor, 2010; Albenberg and Wu, 2014) and negatively affect biodegradable dosage forms that rely on enzymes of the microbiome for drug release. Therefore, co-administration of other drugs may cause inter-individual and intra-individual variability with respect to oral drug delivery and should be considered in oral formulation design, especially for specific disease indications.

### Impact of Gastrointestinal Surgery on Oral Drug Delivery

Surgical resections of the stomach, small intestine or large intestine can significantly affect gastrointestinal anatomy and physiology, as well as the effectiveness of oral dosage forms and drug absorption (Titus et al., 2013; Hua et al., 2015; Hatton et al., 2018). Partial gastric resection or bypass is performed for the treatment of peptic ulcer disease, malignancy, and as a means of weight loss. Although most drugs are minimally absorbed in the stomach, gastric resections and bariatric surgeries can affect gastric emptying and transit time (Titus et al., 2013). For example, vagotomy can delay gastric emptying, whereas resection of the pylorus can accelerate gastric emptying (Titus et al., 2013).

Intestinal resections can be the result of a number of diseases, including in severe IBD, malignancy, and in intestinal malrotation with ischemia. In general, small resections usually pose minimal issues for oral drug delivery, as the remaining intestine can compensate so that no functionality is lost (Kvietys, 1999; Titus et al., 2013). However, when large resections (usually greater than 50%) are performed, there may be profound changes in gastrointestinal function, including motility and drug absorption. For example, shortening of the intestine can reduce the transit distance through the GI tract, which potentially affects the way conventional oral formulations are processed (Kvietys, 1999; Titus et al., 2013). Resection can also significantly change the physiology of the intestinal tract by altering pH, digestion, transit, and nutrient absorption (Spiller et al., 1988; Schmidt et al., 1996; Fallingborg et al., 1998). For example, surgery is one of the main treatments for colorectal cancer which is defined as the development of malignant cells in the colonic epithelium (Patel, 2014; Banerjee et al., 2017). For more advanced disease, a colectomy (surgical procedure to remove all or part of the colon) is required, which will alter the local microenvironment and physiology of the GI tract (Titus et al., 2013). Many IBD patients also undergo surgical resection of intestinal tissues (Byrne et al., 2007). Consequences of these resections include a shortened bowel that may have associated implications for oral dosage form design. This includes altering luminal pH and transit times, impairing regulation of the ileal brake that controls food transit, and reduction of small chain fatty acid digestion (Kvietys, 1999; Titus et al., 2013; Hua et al., 2015).

Similarly, profound changes in gastrointestinal physiology and drug delivery can occur when specific segments of the GI tract are resected. In particular, resection of the terminal ileum alters water absorption and dilutes residual bile acids in the colon, thereby reducing net colonic fatty acid concentrations (Thompson et al., 1998; Gracie et al., 2012). The decrease in fatty acids reduces the ileal brake, which is a nutrient feedback mechanism that slows transit times to allow nutrient absorption (Van Citters and Lin, 1999; Van Citters and Lin, 2006). As fatty acids are the most potent stimulant of the ileal brake, a loss of both fatty acids from digestion and fatty acid receptors from resected tissue lead to a loss of the ileal brake (Lin et al., 2005) and, therefore, cause more rapid intestinal transit and less time for absorption. The terminal ileum is also responsible for bile salt reabsorption and, when removed, can become problematic and is generally manifested by choleretic diarrhea (Titus et al., 2013) that can significantly affect the therapeutic efficacy of conventional oral formulations.

## Conventional Oral Drug Delivery Approaches

The main formulations used for oral drug delivery are liquid dosage forms (such as solutions and suspensions) and solid dosage forms (such as tablets and capsules) (Allen et al., 2011). Because solid dosage forms need to disintegrate and then dissolve the drug before absorption can occur, dissolution rate determines availability of the drug for absorption (Martinez and Amidon, 2002). Manipulating the formulation can control the dissolution rate and where the drug is released in the GI tract for subsequent absorption. Their design is based on exploiting physiological conditions in the GI tract. By using modified formulations, it is possible to improve targeting to three different parts of the GI tract — namely the stomach, the small intestine, and the colon.

### Gastroretentive Drug Delivery Systems

Prolonging the gastric residence time of dosage forms is particularly beneficial for drugs that are predominantly absorbed in the stomach or upper GI tract, or for drugs that suffer from solubility issues in the intestinal fluid (Mandal et al., 2016). This promotes the slow release of drug in the stomach, which subsequently extends the time available for drug dissolution and absorption in the stomach and/or small intestine. The benefit of this approach also includes sustained or controlled release drug delivery, which can reduce fluctuations in systemic drug concentrations as well as increase patient compliance to medications by minimizing the number of doses required (Awasthi and Kulkarni, 2016). Ideally, gastroretentive dosage forms should remain in the stomach for a specific duration and be able to undergo clearance from the body. For example, they should consist of components that are biodegradable or can undergo disintegration to smaller components after a predetermined time period. However, the prolonged nature of the dosage form would mean that immediate ceasing of a drug would be difficult, especially for patients experiencing adverse effects or hypersensitivity reactions.

The formulation approaches for gastroretention have been extensively reviewed (Streubel et al., 2006a; Mandal et al., 2016; Awasthi and Kulkarni, 2016; Tripathi et al., 2019) and include the following: (i) high-density dosage forms that sink into the folds of the antrum; (ii) floating dosage forms over gastric content; (iii) mucoadhesive dosage forms to gastric mucosa; and (iv) expandable dosage forms which expand or swell in the stomach to larger dimensions. Although there have been an extensive number of studies in the literature on gastroretentive dosage forms, the clinical translation of these technologies has not progressed as rapidly. Of these approaches, the floating dosage forms are the most common commercialized gastroretentive drug delivery system (Kumar and Kaushik, 2018). The major considerations for floating dosage forms are the susceptibility of the dosage form to body position (Timmermans and Moes, 1994) and the requirement to maintain a sufficient stomach content to allow an effective separation between the dosage form and the pyloric region (Whitehead et al., 1998; Kagan and Hoffman, 2008).

Expandable dosage forms have garnered particular attention in recent years. Ideally, these dosage forms should be small enough to swallow and be able to rapidly increase in size once in the stomach to prevent premature emptying through the pylorus (Streubel et al., 2006b). The diameter of the pylorus is approximately 12 ± 7 mm (Timmermans and Moes, 1993), which means that the size of the dosage form needs to be larger. It is generally accepted that a diameter >15 mm is required for prolonging gastric retention, especially during the fasted state (Timmermans and Moes, 1993; Bardonnet et al., 2006). The performance of these particular dosage forms is not dependent on the filling state of the stomach. There are several safety issues that need to be assessed for this type of dosage form, including the potential for accumulation of several dosage units in the stomach following multiple administrations, as well as possible occlusion of the esophagus or pylorus (Kagan and Hoffman, 2008).

Mucoadhesive and high-density dosage forms for gastroretention have translational limitations. For example, mucoadhesive dosage forms can be unpredictable regarding the site of adhesion (including the risk of esophageal binding) and can potentially suffer from elimination due to the high mucus turnover rate in the stomach (Rubinstein and Tirosh, 1994; Streubel et al., 2006b). The main disadvantage of high-density dosage forms is that they can be technically difficult to manufacture, generally requiring a large amount of drug due to the progressive decrease in the weight of the matrix as the drug gets released (Rouge et al., 1998; Awasthi and Kulkarni, 2016).

### Regional Drug Targeting in the Small Intestine

Regional targeting of drugs to the small intestine is usually attained with gastroretentive dosage forms, pH-dependent dosage forms, or mucoadhesive dosage forms. Controlled drug delivery formulations with prolonged gastric residence time can be advantageous for drugs that are absorbed in the small intestine, especially those with an absorption window in the upper small intestine (Streubel et al., 2006b). Gastrointestinal transit time through the upper small intestine is rapid, thereby limiting the time available for absorption at this site. The advantages and disadvantages for each of the gastroretentive dosage forms have been discussed above *(refer to “Gastroretentive Drug Delivery Systems”)*.

Formulations that have pH-responsive coatings or matrices are particularly beneficial for drugs that are susceptible to degradation by gastric enzymes or by the acidity of the gastric fluid, as well as for drugs that can cause irritation to the gastric mucosa (Rouge et al., 1996; Thakral et al., 2013; Liu et al., 2017). In particular, enteric-coated solid dosage forms (e.g., tablets and capsules) are commonly used and are available clinically (Thakral et al., 2013; Al-Gousous et al., 2017). An enteric coating is defined as a material, usually a polymer, that forms a barrier over the surface of the dosage form that permits transit through the stomach to the small intestine before the drug is released (Felton and Porter, 2013; Thakral et al., 2013). However, disintegration and absorption from formulations containing enteric coatings or pH-responsive matrices may be erratic, due to the relatively slow dissolution or degradation of the polymers in comparison to the transit time of the formulation through the small intestine (Al-Gousous et al., 2017; Kang et al., 2018). Variability in gastric emptying time can also affect drug release in the small intestine (Al-Gousous et al., 2017). In addition, considerable intra- and inter-individual variability in the pH of the GI tract will affect drug release from pH-dependent dosage forms (Fallingborg et al., 1993; Sasaki et al., 1997; Nugent et al., 2001; Ibekwe et al., 2008; McConnell et al., 2008; Lahner et al., 2009; Brunton et al., 2018).

Mucoadhesive dosage forms, especially intestinal patches, have been investigated to prolong contact with the intestinal mucosa to improve drug absorption (Shen and Mitragotri, 2002; Toorisaka et al., 2012; Banerjee et al., 2016a; Gupta et al., 2016; Banerjee and Mitragotri, 2017). They are also able to protect the drug from degradation during transit in the upper GI tract. Drug release is influenced by formulation factors such as polymer composition, mucosal adhesive strength, drug concentration, drug release rate, and drug release direction (i.e., unidirectional or bidirectional) (Banerjee and Mitragotri, 2017; Homayun et al., 2019). These dosage forms are limited by the fact that they require sufficient binding with the intestinal wall to avoid being washed away by solid boluses of digested food, gastric and intestinal fluids, or by the continuous secretion and turnover of mucus (Banerjee and Mitragotri, 2017; Homayun et al., 2019). Being mucoadhesive in nature, there is a risk of adhesion with other mucosal surfaces following oral administration before entering the small intestine. This may lead to the release of drug into a region where it has minimal absorption capacity or is easily degraded. In addition, specificity in the site of binding in the small intestine, which is already extensive in length and highly convoluted, is also difficult to predict. Adhesion to the proximal region of the duodenum would be most ideal, as the latter regions are exposed to boluses of digested food that are more solid in form, which can more readily detach the patch from the luminal surface (Banerjee and Mitragotri, 2017; Homayun et al., 2019). However, some drugs are known to have preferential absorption sites in the small intestine (Murakami, 2017).

### Regional Delivery of Drugs to the Colon

Colon targeted drug delivery is an active area of research, particularly for the treatment of local diseases affecting the colon, such as IBD and colorectal cancer. Improving the delivery of drugs to the colon not only improves the local effectiveness of therapeutics, but it can also reduce the risk of systemic adverse effects. Three main strategies are commonly used in conventional formulations for the regional delivery of drugs to the colon (Van den Mooter, 2006; Kagan and Hoffman, 2008; Vass et al., 2019): (i) utilization of a pH drop on entry into the colon; (ii) delayed release dosage forms that rely on gastrointestinal transit time; and (iii) exploitation of metabolic capabilities of the colonic microbiome.

#### pH-Responsive Dosage Forms

In general, the first approach uses pH-specific coatings and matrices that are soluble at neutral or slightly alkaline pH to release the drug in the distal part of the small intestine or in the colon. **Table 2** shows some examples of pH-dependent polymer coatings that have been used for the purpose of colonic targeting either alone or in combination, including some methacrylic resins (commercially available as Eudragit^®^) (Khan et al., 1999; Goto et al., 2004; Thakral et al., 2013) and hydroxypropyl methylcellulose (HPMC) derivatives (Nykanen et al., 2001; Gareb et al., 2016). In addition to triggering release at a specific pH range, the enteric coating protects the incorporated active agents against the harsh GI tract environment (e.g., gastric juice, bile acid, and microbial degradation) and can create an extended and delayed drug release profile to enhance therapeutic efficiency (Yang et al., 2002; Van den Mooter, 2006). Targeting the colon with such polymers has proved difficult due to considerable intra- and inter-individual variability in the pH of the GI tract (McConnell et al., 2008), which is also influenced by diet (Ibekwe et al., 2008), disease (Fallingborg et al., 1993; Sasaki et al., 1997; Nugent et al., 2001), and co-administered drugs (Lahner et al., 2009; Brunton et al., 2018). Despite this variability, pH responsive approaches to colonic delivery have been used commercially. For example, mesalazine used for IBD is commercially available as oral tablets coated with Eudragit L-100 (Mesasal^®^ and Colitofalk^®^) or Eudragit S (Asacol^®^).

**Table 2:** Examples of pH-dependent polymer coatings used for colonic targeting.

Polymer	Optimum pH
Eudragit^®^ S-100	7.0
Eudragit^®^ FS 30D	7.0
Eudragit^®^ L-100	6.0
Cellulose acetate phthalate	6.0
Cellulose acetate trimellitate	5.5
Eudragit^®^ L 30D-55	5.5
Eudragit^®^ L 100-55	5.5
Hydroxypropyl methylcellulose phthalate 55	5.5
Hydroxypropyl methylcellulose phthalate 50	5.0
Polyvinyl acetate phthalate	5.0

#### Time-Dependent Dosage Forms

Time-dependent formulations essentially use gastrointestinal transit times as a guide to activate drug release into the colon. These formulations typically rely on the relatively constant transit time through the small intestine, and work on the assumption that a dosage form will spend approximately 6 h in the stomach and small intestine in the fasted state. They are typically composed of hydrophilic polymers (e.g., ethyl cellulose and HPMC) in the coating or matrix that are able to gradually swell over time, which creates a lag phase before releasing the drug (Sangalli et al., 2001; Gazzaniga et al., 2006; Gareb et al., 2016). In particular, drug release from hydrophilic matrices depends on several processes, including swelling of the polymer, penetration of water through the matrix, drug dissolution, drug transport through the swelled polymer, and erosion of the matrix (Colombo et al., 1995; Colombo et al., 2000; Caraballo, 2010). Hydration of the polymer when in contact with aqueous fluids changes the structure of the polymer to form a gel layer, which controls the drug release rate (Caraballo, 2010). Drug release is also influenced by formulation factors related to the polymer (e.g., composition, concentration, distribution, viscosity) and drug (e.g., loading, solubility, particle size) (Siepmann and Peppas, 2001; Miranda et al., 2007; Caraballo, 2010).

The main disadvantage of this approach is the huge variability seen in gastrointestinal transit time in the stomach, small intestine, and colon—with many physiological, pathophysiological, and pharmaceutical factors influencing these parameters (*refer to sections Physiological Factors Influencing Oral Drug Delivery and Pathophysiological Factors Influencing Oral Drug Delivery*). For example, gastric emptying time can be significantly prolonged after eating, which can lead to premature drug release in the small intestine instead of the colon (Ibekwe et al., 2008; Reinus and Simon, 2014). In addition, gastrointestinal transit time can be altered when associated with disease, such as IBD. Colonic transit is typically faster in IBD patients and is likely due to diarrhea, which is typically worse during active disease (Hebden et al., 2000; Podolsky, 2002). This can lead to difficulties in targeting specific regions of the colon with conventional formulations. For example, conventional delayed release formulations have been reported to show asymmetric drug distribution in the colon, with significantly lower drug concentrations in the distal colon and higher drug retention in the proximal colon (Hebden et al., 2000). Therefore, transit time may not be a reliable approach for targeted drug delivery in the colon when associated with some diseases.

#### Biodegradable Dosage Forms

The consistently high levels of resident bacteria in the colon have been exploited for colon-specific drug delivery and is considered a much more reliable factor (McConnell et al., 2008). Numerous enzymes are produced by the colonic bacterial flora, such as polysaccharidases, azoreductases, and glycosidases (Scheline, 1973; Cummings and Macfarlane, 1991; Rubinstein, 2000), and have been utilized in drug delivery approaches. For example, biodegradable polymers in coatings and/or matrix formulations have been used for regional drug targeting in the colon. In particular, polysaccharide-based systems have shown promising results, with non-starch polysaccharides being commonly used (Hovgaard and Brondsted, 1996; Rubinstein, 2000; Shah et al., 2011). Non-starch polysaccharides are more resistant to digestion and absorption in the small intestine but are metabolized in the large intestine. These polymers are generally hydrophilic and are able to hydrate and swell during transit through the GI tract (hence they are also exploited in time-dependent dosage forms). The hydrated layers allow the penetration of colonic bacteria and enzymes, which lead to degradation and drug release within an acceptable duration (Van den Mooter, 2006; Shah et al., 2011). It should be noted that most of these polymers are strongly hydrophilic, which can lead to premature drug release before the colon is reached (Hovgaard and Brondsted, 1996; Van den Mooter, 2006; Patel, 2015). Premature drug release can also occur with the inter- and intra-individual variability in gastrointestinal transit times (Coupe et al., 1991; Watts et al., 1992; Timmermans and Moes, 1994; Rao et al., 2004; Kagan and Hoffman, 2008; Reinus and Simon, 2014; Brunton et al., 2018). Therefore, few have reached the clinic due to lack of specificity in drug release. Chemical modification of polysaccharides or combining them with other conventional hydrophobic polymers have been investigated as a way to increase their hydrophobicity. It should be noted that a balance between hydrophilic and hydrophobic properties of the polysaccharides is required. Those that have low water solubility may have better capability for drug retention but can suffer from issues with low degradation (Hovgaard and Brondsted, 1996; Shah et al., 2011).

Similarly, azoreductase activity of colonic bacteria has been extensively studied for colon-targeting systems, especially in the development of prodrugs (Rafii et al., 1990; Oz and Ebersole, 2008; Marquez Ruiz et al., 2012). Prodrugs essentially rely on the enzymatic activity of colonic bacteria to break down an inactive precursor and release the active drug moiety. This approach is usually used to improve physicochemical properties of drugs (e.g., solubility, permeability, and stability) and/or to target drug release to a specific site in the GI tract. This occurs with the prodrugs of 5-aminosalicylic acid (5-ASA), such as sulfasalazine and olsalazine, which are used in the treatment of IBD (Oz and Ebersole, 2008). For example, sulfasalazine has low absorption in the upper GI tract and is cleaved by azoreductases of the microflora in the colon to release the active 5-ASA moiety, which is thought to have local actions in the colon. Azoreductase enzymes are largely produced by anaerobes present in the proximal part of the large intestine and onwards (Rafii et al., 1990; Oz and Ebersole, 2008; Marquez Ruiz et al., 2012).

Pathological changes in the microflora can occur in diseases, such as IBD and gastrointestinal infections, as well as with the use of drugs (e.g., antibiotics) (Linskens et al., 2001; Sartor, 2010; Hua et al., 2015). This can affect the composition and diversity of bacterial species and, therefore, the secretion of enzymes that are important in triggering drug release for microbial-dependent drug delivery systems. In addition, considerable loss of biodegradable dosage forms may occur in the case of diarrhea, due to insufficient time for activation or drug release (Sartor, 2010; Albenberg and Wu, 2014).

#### Combination of Strategies

To circumvent the issues with variability in gastrointestinal physiology, a combination of colon-targeting strategies has been utilized in conventional formulations. For example, both pH and time-dependent strategies are commonly used to improve drug delivery to the colon (Zema et al., 2007; Talaei et al., 2013; Patel, 2015). For example, one of the first formulations of this type was Pulsincap^®^ (Wilding et al., 1992; Stevens et al., 2002; Jain et al., 2011; Patel et al., 2011). It consists of a capsule, half of which is enteric-coated and the other half is non-disintegrating. The enteric coat protects against gastric acid and avoids the problem of variable gastric emptying. This coat dissolves on entering the small intestine, revealing a hydrogel plug that then starts to swell. Timing of drug release is governed by the amount of hydrogel, in that the hydrogel plug is ejected from the bottom half of the capsule with extensive swelling.

In addition, Entocort^®^ EC is another example of a dosage form that uses a combination of pH and gastrointestinal transit time (McKeage and Goa, 2002; Edsbacker and Andersson, 2004). The dosage form contains ethyl cellulose-based granules that are approximately 1 mm in size and contain budesonide (corticosteroid). Each granule is coated with Eudragit^®^ L, which is a pH-dependent coating that dissolves at pH >5.5 to allow drug release in the ileum and ascending colon. The ethyl cellulose granules then ensure time-dependent drug release in the colon. This multiparticulate formulation is indicated for colonic inflammation, particularly for IBD (McKeage and Goa, 2002; Edsbacker and Andersson, 2004). The combination approach has shown promising results in improving drug release in the colon and reducing premature drug release in the upper GI tract. However, it can still suffer from the intra- and inter-individual variability that can occur with each of these gastrointestinal parameters.

## Nanoparticulate Oral Drug Delivery Approaches

The development of novel gastrointestinal drug delivery systems has gained increasing interest, due to the inconsistent efficacy and inter-patient variability of conventional approaches that mostly rely on non-stable parameters in the GI tract. In particular, nanoparticulate dosage forms have shown promising results in drug delivery compared to conventional single-unit dosage forms. These formulations contain a number of separate nanoparticle subunits in which the dose of the drug is distributed across. This allows them to overcome the challenges faced by single-unit dosage forms, such as unpredictable disintegration and dissolution, nonspecific drug release, dose dumping, and stability issues in the GI tract (Talaei et al., 2013; Hua et al., 2015; Shahdadi Sardo et al., 2019). Nanoparticles have a larger surface-area-to-volume ratio, which provides a greater surface area for interaction with the mucosal surface and for the solubilization of drugs. Nanoparticulate dosage forms have shown the following advantages for gastrointestinal drug delivery, owing to their smaller size: (i) easier transport through the GI tract; (ii) more uniform distribution and drug release; (iii) increase in residence time of particles in the GI tract, even when colonic motility is increased in diarrhea; (iv) improved uptake into mucosal tissues and cells; and (v) specific accumulation to the site of disease, such as inflamed tissues (Hua et al., 2015; Moss et al., 2018; Reinholz et al., 2018). Nanoparticles generally undergo cellular uptake *via* the transcellular pathway in the GI tract (Yu et al., 2016; Reinholz et al., 2018). Translocation of nanoparticles can also occur by paracellular transport and persorption through gaps or holes at the villous tips (Hillyer and Albrecht, 2001; des Rieux et al., 2005).

### Nanoparticulate Dosage Forms for Gastric Delivery

There are limited studies that have investigated the use of nanoparticulate formulations for gastric drug delivery. A major issue is the rapid passage of nanoparticles through the stomach to the intestine due to their small particle size (Sarparanta et al., 2012). Size is an important parameter for gastroretentive dosage forms, with particles less than 7 mm in diameter being efficiently evacuated (Timmermans and Moes, 1993; Bardonnet et al., 2006). However, the advantage of nanoparticulate formulations is the dispersion of the drug across multiple subunits and, therefore, the distribution of multiple subunits throughout the stomach. This avoids the limitations of single-unit dosage forms. The size of the nanoparticles may also improve mucosal interaction, with the potential for cellular uptake and/or close interaction for efficient drug delivery. The delivery of high drug concentrations in the stomach is particularly beneficial for the treatment of local diseases such as gastritis, gastric ulcer, and bacterial infections (e.g., *Helicobacter pylori*), as well as for drugs that have better absorption in the stomach (Rouge et al., 1996; Mandal et al., 2016).

To address the potential for rapid clearance from the stomach, studies have incorporated gastroretentive strategies to nanoparticulate formulations, especially mucoadhesive (Umamaheshwari et al., 2004; Ramteke et al., 2008; Ramteke and Jain, 2008; Jain et al., 2009; Ramteke et al., 2009; Sarparanta et al., 2012; Ngwuluka et al., 2015; Jain et al., 2016; Sunoqrot et al., 2017) and high-density systems (Ngwuluka et al., 2015; Sharma et al., 2018). The studies have shown promising results with regard to gastric retention and/or mucoadhesion in both *in vitro* and *ex vivo* experiments. However, extrapolation of these results to animals and humans is difficult, as there are a number of significant physiological and pathophysiological factors that affect gastric drug delivery. For example, the success of gastroretentive dosage forms has been limited due to high gastric motility and rapid mucus turnover. The stomach content is also highly hydrated, which can affect the adhesion of many mucoadhesive polymers (Pawar et al., 2011; Sunoqrot et al., 2017).

Initial *in vivo* biodistribution studies of nanoparticulate dosage forms have demonstrated prolonged gastroretention of up to 3 h in animals that have been fasted (Sarparanta et al., 2012). Although this parameter was not assessed in other *in vivo* studies on nanoparticles, those on microparticulate dosage forms have shown prolonged gastric retention of over 8 h in the fasted state (Hao et al., 2014). The difference is likely due to the gastroretentive strategy applied to the particles as well as the animal species used in the study. In rodents, the stomach is divided into the forestomach where ingested material is stored, and the glandular stomach where digestion continues (Gartner, 2002). Sarparanta et al. (Sarparanta et al., 2012) reported that the majority of the orally administered mucoadhesive nanoparticles were found to be mixed with material that the animals had ingested during the experiment (e.g., hair and bedding chips) in the forestomach. This is likely to interfere with the adhesion of the nanoparticles with the mucosa. However, sheets of nanoparticles and nanoparticle aggregates were found strongly adhered to the mucosa in the glandular stomach.

Most of the *in vivo* efficacy studies have been focused on using nanoparticulate formulations for treating *Helicobacter pylori* infection. Efficacy of drug-loaded nanoparticles have been demonstrated in *Helicobacter pylori* infected animals, even with once daily dosing, due to their mucoadhesive properties (Umamaheshwari et al., 2004; Ramteke et al., 2008; Jain et al., 2009; Ramteke et al., 2009). The results have been promising, however further *in vivo* investigations are required in more clinically relevant animal models to determine the translatability and reproducibility of nanoparticulate formulations for gastric drug delivery. It would also be important to understand the performance of nanoparticulate dosage forms under both fed and fasted conditions. For effective clinical translation, it is likely that the nanoparticles will also need to be loaded into a capsule that is able to dissolve rapidly in the stomach. This will ensure stability during transit in the oral cavity and esophagus, as well as maximal release of nanoparticles in the stomach.

### Nanoparticulate Dosage Forms for Small Intestinal Delivery

Nanoparticulate formulations have been applied to the regional targeting of drugs in the small intestine to improve both local and systemic absorption. This is particularly beneficial for drugs that have poor solubility in the small intestine or are unstable in the harsh gastric environment (Lundquist and Artursson, 2016). By increasing the bioavailability of drugs into the small intestine, nanoparticles can be designed to: (i) trigger drug release in the lumen for subsequent absorption; (ii) adhere to the mucosal surface for effective drug release and absorption; (iii) enhance mucosal uptake of intact nanoparticles with subsequent drug release for local or systemic absorption; or (iv) enhance mucosal uptake and absorption of intact nanoparticles into the systemic circulation. There are a number of studies which have reported enhanced systemic absorption of drugs in the small intestine from nanoparticulate formulations (Bargoni et al., 1998; Fonte et al., 2011; Zhang et al., 2011; Reix et al., 2012; Zhang et al., 2013a; Tariq et al., 2016; Ahmad et al., 2018; Prajapati et al., 2018). However, in the majority of cases, the specific mechanism of action was not elucidated.

The mucosal uptake of intact nanoparticles is the most challenging, as the nanoparticles would need to cross multiple cellular barriers after penetrating the mucus layer (Reinholz et al., 2018). For example, nanoparticles would need to cross the intestinal epithelium to reach the lamina propria and then traverse a layer of endothelial cells of the blood vessels for systemic delivery. Nanoparticles can cross the intestinal epithelium *via* three main pathways — paracellular transport (between cells through tight junctions), transcellular transport (through the interior of cells with subsequent exocytosis), and M-cell-mediated transport (Yu et al., 2016; Reinholz et al., 2018). The advantages and limitations of each pathway are summarized in **Table 3**.

**Table 3:** Summary of the main pathways that nanoparticles can take to cross the intestinal epithelium (Yu et al., 2016; Reinholz et al., 2018).

**Paracellular**	Transport through the intercellular space between intestinal epithelial cells (enterocytes)
	Intercellular spaces have an aqueous environment and rely on passive transport
	*Limitations*
	Passage of nanoparticles is restricted by the narrow tight junction space (0.3 to 20 nm)
	Potential for toxicity with the passage of other gastrointestinal content in the chyme
**Transcellular**	Transport through epithelial cells (enterocytes) by transcytosis, which includes endocytosis, intracellular trafficking, and exocytosis
	Enterocytes represent 90–95% of the cells lining the GI tract
	Nanoparticles can potentially undergo indirect transport to the systemic circulation *via* the hepatic portal system or direct transport to the systemic circulation *via* the intestinal lymphatic system
	*Limitations*
	Internalized nanoparticles are usually transported to lysosomes that contain a variety of enzymes for degradation
	Enterocytes have enzymes in the microvilli of the brush border membrane and within the glycocalyx
	Mucus layer and glycocalyx of enterocytes are thicker compared to M cells
**M-cell-mediated**	Transport through M cells (microfold cells) by transcytosis, which includes endocytosis, intracellular trafficking, and exocytosis
	M cells are mainly localized in Peyer's patches in the small intestine and have reduced intracellular enzymatic activity
	Mucus layer and glycocalyx of M cells are considerably thinner compared to enterocytes, allowing easier access
	Nanoparticles can potentially be captured by macrophages and dendritic cells in the Peyer's patches (beneficial for the development of oral vaccinations) or undergo passive lymphatic targeting followed by systemic drug delivery
	*Limitations*
	Absorption of nanoparticles is restricted due to the low proportion of M cells (~1%) in the intestinal epithelium
	Cellular uptake can be low due to a lack of specificity of nanoparticles towards M cells

#### Paracellular Transport

The passage of nanoparticles by paracellular transport is restricted by the narrow tight junction space, which can range from 0.3 nm to 20 nm, depending on the state (Madara, 1998; Camenisch et al., 1998; Acosta, 2009; Chen et al., 2013). Incorporation of charged polymers has been investigated as a means to reversibly open tight junctions and improve drug delivery across the intestinal epithelial barrier. For example, chitosan (cationic polymer) has been reported to facilitate the paracellular transport of nanoparticles (Zhang et al., 2014a; Liu et al., 2016). The rapid and reversible absorption-enhancing effect of chitosan was suggested to be due to changes in intracellular pH caused by the activation of a chloride-bicarbonate exchanger, thereby resulting in the opening of the tight junctions (Rosenthal et al., 2012). Peptides that have the capability of modulating the degree and kinetics of tight junctions have also demonstrated enhanced paracellular transport of drugs (Taverner et al., 2015). However, the size restriction needed for effective paracellular transport would limit most nanoparticulate formulations as well as the potential for toxicity with the passage of other gastrointestinal content in the chyme (Reinholz et al., 2018).

#### Transcellular Transport

Transcellular transport of nanoparticles across enterocytes is considered the most promising pathway for small intestinal drug delivery, owing to the large representation of these epithelial cells lining the GI tract (Reinus and Simon, 2014). Nanoparticles can then potentially undergo indirect transport to the systemic circulation *via* the hepatic portal system or direct transport to the systemic circulation *via* the intestinal lymphatic system. The intestinal lymphatic systemic can be targeted *via* lacteals, which are lymphatic capillary vessels in the villi of the small intestine (Reinus and Simon, 2014; Managuli et al., 2018). There are several challenges with this particular pathway, including the following: (i) the thick mucus layer overlaying the enterocytes; (ii) the thick glycocalyx coating the surface of the enterocytes; (iii) the luminal enzymes; and (iv) the enzymes in the microvilli of the brush border membrane and within the glycocalyx (Kyd and Cripps, 2008; Lundquist and Artursson, 2016; Yu et al., 2016). Together, these barriers help to prevent pathogens and potential toxins in the gastrointestinal content from entering the body.

Although intestinal barriers play a protective role in the body, they can also restrict the uptake of nanoparticulate formulations by enterocytes, which means that most of the nanoparticles are degraded or eliminated from the body (Yu et al., 2016). Nanoparticles that are internalized within enterocytes face additional challenges that restrict them from undergoing transcytosis. In particular, they are usually transported to lysosomes for degradation. This typically involves the transport of nanoparticles in endosomes, which can eventually fuse with the cell membrane for exocytosis or fuse with lysosomes for degradation (Hofmann et al., 2014; Hu et al., 2015). Lysosomes are intracellular vesicles with an acidic pH of 4.5–5 (Mindell, 2012) and contain a variety of enzymes that have a physiological role in degrading or recycling foreign molecules or cellular compounds (Saftig and Klumperman, 2009). Entrapment and degradation of nanoparticles within lysosomes prevent exocytosis at the basolateral membrane, which affects the efficacy of nanoparticulate formulations (Yu et al., 2016; Reinholz et al., 2018).

Several approaches have been utilized to improve the delivery and transcytosis of nanoparticles across enterocytes in the small intestine. The main parameters are particle size, nanoparticle composition, and surface modification. Studies have demonstrated an inverse correlation between particle size and cellular uptake, with improved uptake with smaller nanoparticles (50 nm > 200 nm > 500 nm > 1000 nm) (Desai et al., 1996; Bannunah et al., 2014; Banerjee et al., 2016b). Following uptake into enterocytes and subsequent basolateral secretion into the interstitial space, nanoparticle size can potentially influence whether they are selectively taken up by the lymphatic system or hepatic portal system, with larger particles having a preference for the lymphatic system (Griffin et al., 2016). In addition, a variety of materials have been used to construct nanoparticles, including lipids and polymers. Further stability studies are required to determine the *in vivo* small intestinal bioavailability of these nanoparticles following oral administration. For example, the harsh enzymatic environment might be particularly detrimental to lipid-based nanoparticles due to lipolysis (Beloqui et al., 2016; Hu et al., 2016; Shreya et al., 2018). Incorporation of additional strategies may be required to protect nanoparticles from premature degradation in the GI tract (Makhlof et al., 2011a).

The effect of physicochemical parameters, other than particle size, is only beginning to be understood. Only a few studies have investigated the effect of surface charge, hydrophobicity, and shape on the bioavailability and absorption of nanoparticles after oral administration. In general, cationic nanoparticles showed enhanced uptake and transport by enterocytes compared to those with an anionic or neutral charge (Bannunah et al., 2014; Hellmund et al., 2015; Du et al., 2018) as well as significantly increased oral bioavailability *in vivo* (Du et al., 2018). Importantly, the cationic nanoparticles were not only internalized by the intestinal epithelial cells, but they were also transported through these cells into the lamina propria (Du et al., 2018). In addition, coating the surface of nanoparticles with polyethylene glycol (PEG) to create a hydrophilic surface chemistry minimized strong interaction with the mucus constituents and increased particle translocation through the mucus as well as mucosa (Maisel et al., 2015; Du et al., 2018). With regard to nanoparticle shape, initial studies have demonstrated higher cellular uptake and transcytosis of rod-shaped nanoparticles compared to sphere-shaped nanoparticles (Banerjee et al., 2016b). Nanorods also exhibited significantly longer retention time in the GI tract (especially in the jejunum and ileum) compared to nanospheres, which allowed more time for intestinal absorption (Li et al., 2017). They showed improved penetration into the space between the intestinal villi, with only low absorption of intact nanoparticles (Li et al., 2017).

Improvements in the translocation of nanoparticles within enterocytes have also been achieved with ligand-mediated active targeting. This strategy involves the conjugation of ligands to the surface of nanoparticles and exploits cell-specific differences or disease-induced changes in the expression of receptors, proteins, and adhesion molecules on the surface of tissues (Hua et al., 2015; Sercombe et al., 2015). Interactions between targeting ligands and specific receptors expressed at the site of action are expected to improve bioadhesion of the carrier to specific cells and increase the extent for cellular uptake. Various receptors expressed on the surface of enterocytes have demonstrated improved uptake and transcytosis of nanoparticles, with improve systemic bioavailability and therapeutic efficacy of encapsulated therapeutics (Zhang and Wu, 2014; Griffin et al., 2016). This includes the conjugation of ligands to the surface of nanoparticles that are specific for the following receptors—vitamin B12 (Chalasani et al., 2007), folate (Anderson et al., 2001; Ling et al., 2009; Jain et al., 2012), biotin (Zhang et al., 2014b), and lectins (Zhang et al., 2005; Yin et al., 2006; Zhang et al., 2006b; Makhlof et al., 2011b). Vitamin B12 ligand-mediated transport is limited by the relatively slow uptake of vitamin B12 in the GI tract as well as restricted site for absorption in the distal ileum (Hamman et al., 2007; Zhang and Wu, 2014). In addition, lectins can show nonspecific interactions with the mucus layer of the intestinal epithelium (Irache et al., 1994; Cornick et al., 2015; Managuli et al., 2018) and can have toxicity and stability issues (Zhang and Wu, 2014).

Enhancing the transcytosis of intact nanoparticles across enterocytes is a promising strategy to improve the systemic delivery of drugs that have poor stability or solubility in the GI tract. However, further studies are required to determine the optimal nanoparticulate design that provides translatable and reproducible outcomes in humans. As the small intestine is the target for these nanoparticulate formulations, considerations should also be given to the stability of the nanoparticles during transit in the upper GI tract.

### M-Cell-Mediated Transport

Uptake of nanoparticles by M cells (microfold cells), which are mainly localized in Peyer's patches in the small intestine, have become attractive targets for drug delivery. M cells are specialized epithelial cells of the gut-associated lymphoid tissues (GALT) that have a sentinel role for the intestinal immune system by transporting luminal antigens through the follicle-associated epithelium to the underlying immune cells (Miller et al., 2007). The M-cell-mediated pathway has been exploited for nanoparticle drug delivery, as M cells have the advantages of reduced intracellular enzymatic activity as well as a considerably thinner mucus layer and glycocalyx in comparison to enterocytes (Frey et al., 1996; Kyd and Cripps, 2008). These factors promote easier access and intracellular transport. There are two main pathways following uptake into M cells: (i) nanoparticles can be captured by macrophages and dendritic cells in the Peyer's patches, which is beneficial for the development of oral vaccinations (Singh et al., 2015; Yu et al., 2019); and (ii) nanoparticles can undergo passive lymphatic targeting followed by systemic drug delivery (Cavalli et al., 2003; Joshi et al., 2014; Joshi et al., 2016; Managuli et al., 2018). However, the absorption of nanoparticles by M cells is limited due to the low proportion of M cells (~1%) in the intestinal epithelium. In addition, cellular uptake can be low due to a lack of specificity of nanoparticles towards M cells (des Rieux et al., 2006; Yu et al., 2016).

Studies have focused on determining the physicochemical characteristics of nanoparticles for optimal uptake by M cells. In general, nanoparticles larger than 5 µm are taken up by M cells but remain entrapped in Peyer's patches, whereas those smaller than 1 µm are taken up by M cells and transported through the efferent lymphatics within macrophages (Eldridge et al., 1989; Eldridge et al., 1990; Managuli et al., 2018). In addition, non-ionic nanoparticles composed of hydrophobic constituents have better uptake by M cells in comparison to hydrophilic and charged nanoparticles (Bargoni et al., 1998; Shakweh et al., 2004; Managuli et al., 2018).

Active targeting strategies have also been applied to improve specificity of targeting to M cells. Major ligands that have been conjugated to the surface of nanoparticles for targeting Peyer's patches include mannose receptor binding ligands (Fievez et al., 2009; Singodia et al., 2012; Youngren et al., 2013; De Coen et al., 2016), lectin-based ligands (Foster et al., 1998; Clark et al., 2000; Clark et al., 2001; Manocha et al., 2005; Chionh et al., 2009), and integrin specific ligands (Frey et al., 1996; Fievez et al., 2009). It should be noted that there are limited M cell specific targets that have been identified (Zhao et al., 2014), with many also being expressed on other elements in the GI tract. For example, mannose receptors are localized on the apical surface of enterocytes (Fievez et al., 2009; Managuli et al., 2018). In addition, lectins can interact with the carbohydrate residue in the mucus layer of the intestinal epithelium (Irache et al., 1994; Diesner et al., 2012; Cornick et al., 2015; Managuli et al., 2018). Of the targets identified, integrin specific ligands appear to be the most promising target for M cells due to its specificity. However, further *in vivo* studies are required to determine the translatability of these platforms for clinical use. Common laboratory animal species have been reported to have significantly higher density of Peyer's patches in the intestine compared to humans (Kararli, 1995). This should be taken into account to avoid an overestimation of the nanoparticle transport capacity in humans (Lundquist and Artursson, 2016).

### Nanoparticulate Dosage Forms for Colon Delivery

The use of nanoparticulate formulations have demonstrated promising results for colonic drug delivery (Hua, 2014; Hua et al., 2015; Zhang et al., 2017). Reduction in particle size can also enhance targeting and uptake within diseased tissue in the colon. For example, nanoparticles can promote enhanced and selective delivery of drugs into inflamed colonic tissue by exerting an epithelial enhanced permeability and retention (eEPR) effect (Collnot et al., 2012; Xiao and Merlin, 2012), as well as allowing preferential uptake by immune cells that are highly increased in inflamed tissue (Lamprecht et al., 2005a). In addition, nanoparticles are able to avoid rapid carrier elimination that occurs in diarrhea, as these smaller particles are readily taken up into inflamed tissue and cells (Beloqui et al., 2013). When compared to conventional formulations, nanoparticulate formulations have been demonstrated to have improved or similar therapeutic efficacy at lower drug concentrations (Hua et al., 2015).

#### Basic Physicochemical Strategies for Colon Delivery

Nanoparticulate formulations have been designed to passively or actively target the colon. With regards to the ideal particle size for targeting capability in the colon, there have been varying results (Hua et al., 2015). In healthy rats and rats with induced colitis, it was observed that 100 nm particles showed significantly increased accumulation in inflamed colon in comparison to healthy animals (Lamprecht et al., 2001a). Interestingly, initial studies in humans with IBD demonstrated that microparticles (3 µm) had better bioadhesion and accumulation in the inflamed rectal mucosal wall as well as less propensity for systemic absorption (Schmidt et al., 2013). Nanoparticles (250 nm), however, were translocated to the serosal compartment of IBD patients, possibly leading to systemic absorption (Schmidt et al., 2013). Importantly, the total fraction of particles penetrating the rectal mucosa was relatively low in the study (Schmidt et al., 2013). Further studies are required to determine the reason for the difference in particle size response in animals compared to humans.

Although passive targeting, through modifying particle size, enables prolonged retention and improved permeability of nanoparticles, there have been contradictory findings with regards to specificity to diseased versus healthy tissue in the colon (Lamprecht et al., 2005a; Wachsmann et al., 2013). Modification of the surface charge of nanoparticles has been investigated to improve mucosal retention and targeting to diseased tissue. For example, cationic systems are generally considered mucoadhesive, as they adhere to the mucosal surface within inflamed tissue due to the interaction between the negatively charged intestinal mucosa and the positively charged carrier (Liu et al., 2005; Thirawong et al., 2008; Han et al., 2012; Niebel et al., 2012; Coco et al., 2013; Lautenschlager et al., 2013). Colonic mucins have a negative charge since their carbohydrates are substituted with a number of sialic acid and sulfate residues (Larsson et al., 2009; Antoni et al., 2014). In contrast, anionic delivery systems are considered bioadhesive, as they preferentially adhere to inflamed tissue *via* electrostatic interaction with the higher concentration of positively charged proteins (Lamprecht et al., 2001b; Jubeh et al., 2004; Meissner et al., 2006; Beloqui et al., 2013). In particular, high amounts of eosinophil cationic protein and transferrin have been observed in the inflammatory tissue of the colon in IBD patients (Carlson et al., 1999; Peterson et al., 2002; Tirosh et al., 2009). Anionic nanoparticles are able to interdiffuse among the mucus network due to less electrostatic interaction with the mucus in comparison to cationic nanoparticles, which can suffer from immobilization following binding to the mucus (Hua et al., 2015).

Similarly, PEGylated nanoparticles have been demonstrated to improve particle translocation through the mucus as well as mucosa (Tobio et al., 2000; Vong et al., 2012; Lautenschlager et al., 2013). The hydrophilic surface has also been shown to accelerated drug delivery into the leaky inflamed intestinal epithelium (Lautenschlager et al., 2013). Both surface charge and PEGylation are promising pharmaceutical strategies for mucosal targeting, however it is likely that additional colon-specific pharmaceutical strategies are needed to localize the nanoparticles in the colon following oral administration and to further improve targeting to diseased tissue (Hua et al., 2015). It should be noted that there have been conflicting results on the effect of surface charge on colonic targeting, with results mainly based on *ex vivo* tissue binding studies or *in vivo* studies following rectal administration (Hua et al., 2015). There is also a potential for electrostatic interactions and subsequent binding of charged nanoparticles with other charge-modifying substances (e.g., soluble mucins and bile acids) during gastrointestinal transit following oral administration (Hua et al., 2015).

#### Colon-Specific Pharmaceutical Strategies

Colon-specific pharmaceutical strategies are likely required to improve nanoparticle accumulation, retention, and drug release in the colon, as well as minimize drug release in the upper GI tract. Colon-specific approaches can be applied to single-unit dosage forms (e.g., capsules) that are loaded with nanoparticles or applied to each of the individual nanoparticle subunits. The latter approach has been investigated in a number of studies, whereby nanoparticles are modified with components that are sensitive to pH, enzymes, reactive oxygen species (ROS), and overexpressed receptors (Hua et al., 2015). For example, pH-dependent nanoparticulate formulations typically involve coating nanoparticles with pH-sensitive biocompatible polymers to trigger drug release in the colon and protect the incorporated active agents against the harsh gastrointestinal environment in the upper GI tract (Lamprecht et al., 2005b; Makhlof et al., 2009; Kshirsagar et al., 2012; Ali et al., 2014; Beloqui et al., 2014). Although preclinical studies of pH-dependent carriers for colon targeting have been promising, a major concern has been the inherent intra-individual and inter-individual variability of pH and emptying times from the GI tract as well as the change in luminal pH due to disease state.

Biodegradable nanoparticulate formulations take advantage of the consistently high levels of resident bacteria and enzymes in the colon to trigger drug release (Bhavsar and Amiji, 2007; Moulari et al., 2008; Laroui et al., 2010; Kriegel and Amiji, 2011; Kriegel and Amiji, 2011; Laroui et al., 2014a; Xiao et al., 2014). These factors are known to be more consistent to allow efficient colon-targeted drug delivery. Biodegradable polymers have been used in the coatings or matrix of the nanoparticles, including poly-lactic acid (PLA), poly(lactic-co-glycolic acid) (PLGA), and chitosan. In addition, nanoparticles have also been embedded in hydrogel matrices containing polymers that have been shown to be specifically degraded by enzymes in the colon (Laroui et al., 2010; Laroui et al., 2014a; Laroui et al., 2014b; Xiao et al., 2014). Hydrogels are dosage forms that provide a platform for protecting therapeutics through the GI tract and can achieve site-specific delivery by including polymers that exploit fundamental physiological changes (Sharpe et al., 2014). As previously discussed for conventional formulations, biodegradable polymers can suffer from premature drug release or burst release based on their hydrophilicity and solubility in the upper GI tract.

Redox-based nanoparticulate formulations have shown promise for enhancing drug accumulation at sites of colonic inflammation (Wilson et al., 2010). They are able to target diseased tissue of the colon by taking advantage of the abnormally high levels of ROS that are produced at the sites of inflammation to trigger drug release. For example, 10- to 100-fold increase in mucosal ROS concentrations have been reported in biopsies taken from ulcerative colitis patients (Simmonds et al., 1992; Lih-Brody et al., 1996). These were found to be confined to sites of disease and correlated with disease progression (Simmonds et al., 1992; Lih-Brody et al., 1996). The high concentration of ROS is typically generated by activated phagocytes (Mahida et al., 1989). Although there are very few studies available, the initial *in vivo* results have demonstrated localization and efficacy of these nanoparticles to sites of intestinal inflammation in mice with colitis following oral administration (Wilson et al., 2010).

Ligand-mediated active targeting is another promising strategy to enhance drug accumulation and uptake to sites of disease within the colon. This includes the conjugation of ligands to the surface of nanoparticles that are specific for the following — macrophage receptors (e.g., mannose receptors and macrophage galactose-type lectin) (Coco et al., 2013; Xiao et al., 2013; Zhang et al., 2013b; Laroui et al., 2014b), intercellular adhesion molecule-1 (ICAM-1) (Mane and Muro, 2012), transferrin receptors (Harel et al., 2011), and glycoprotein CD98 (Xiao et al., 2014). Additional *in vivo* studies are required to evaluate the efficacy and stability of different targeting ligands and formulations in animal models of colitis (Hua et al., 2015). Commonly used targeting moieties include peptides and monoclonal antibodies, which have been shown to have high targeting specificity and potential mucopenetrative properties (Saltzman et al., 1994). However, oral administration of antibody and peptide-based formulations can suffer from degradation by gastric acid and enzymes in the GI tract. Therefore, further formulation design may be needed for effective oral administration.

## Considerations for Translational Development

Significant advances in the development of oral formulations to improve the regional targeting of drugs in the GI tract have been reported in the literature. However, very few of them have translated to the clinical phase, which is likely due to a combination of biological and pharmaceutical factors. Understanding the relationship between biology and pharmaceutics are important determinants for the successful translation of new formulations (Hua et al., 2018). This includes understanding the effect of physiology and/or pathophysiology on the distribution, retention, disintegration, and release of drugs from oral dosage forms in the GI tract, as well as correlation with *in vivo* behavior (e.g., efficacy and safety) in animals and humans. Differences in the anatomy and/or physiology of the animal species used in *in vivo* studies compared to humans should also be taken into account when evaluating new formulations (Kararli, 1995; Hatton et al., 2015). Considerations should also be given to physiological heterogeneity in the GI tract of both healthy patients and those with specific pathological conditions (Titus et al., 2013; Hua et al., 2015; Hatton et al., 2018; Hatton et al., 2019).

For innovative platforms, such as nanoparticles, safety of the different carriers following uptake needs to be evaluated further. For example, there has been limited studies focused on the toxicology of nanoparticles in the GI tract of humans — this is likely to vary according to the size and composition of the particles (Bergin and Witzmann, 2013; Talkar et al., 2018; Vita et al., 2019). Preclinical studies should be conducted under appropriate blinding and randomization to reduce bias. In addition, assessment against proper controls, including the gold standard treatment and not just free drug solution, is required to determine the potential place in therapy of the innovative platform (Hua et al., 2018). These factors are currently lacking in many published studies, which makes it difficult to assess clinical translatability of the results. Considerations should also be given to the “final product” for clinical use. Nanoparticles can either be delivered as an oral liquid suspension or loaded into solid-dosage forms (e.g., capsules). Depending on the target region in the GI tract, pharmaceutical strategies may need to be incorporated to protect the nanoparticles from premature interaction or degradation during transit. For example, coating capsules or nanoparticles with pH sensitive polymers.

Furthermore, the complexity in the design and development of new formulations should be minimized as much as possible for clinical translation to be justified (Hua et al., 2015; Hua et al., 2018). Platforms that require complex and/or laborious synthesis procedures generally have limited clinical translation potential, as they can be quite problematic and costly to pharmaceutically manufacture on a large scale. Other considerations include availability of materials and industrial equipment, insufficient batch-to-batch reproducibility to set specifications, and overall cost of dosage form development (Hua et al., 2018). Last but not least, there needs to be a clear benefit of efficacy and/or safety with any new oral formulation compared to clinically available dosage forms.

## Conclusion

The oral route of administration is the most preferred route by patients for gastrointestinal drug delivery. However, the performance of the dosage forms and drug absorption are highly dependent on the physiology of the GI tract. Gastrointestinal physiology is complex and can display both large intra- and inter-individual variability. Attempts to overcome these issues have focused on improved understanding of the physiology of the GI tract in both healthy and diseased states. Innovative pharmaceutical approaches are also being explored to improve regional drug targeting in the GI tract, with the majority still in the infancy stages of translational development. For example, the use of multiparticulate dosage systems, such as nanoparticles, has shown promising results in improving gastrointestinal drug delivery compared to single-unit dose formulations. Effective translation will depend on rational dosage form design to enable improvements in gastrointestinal drug delivery for the treatment of both systemic diseases and local gastrointestinal diseases.

## Author Contributions

SH was involved in conception of the idea for the review, drafted the manuscript, and approved the final version of the manuscript.

## Conflict of Interest

The author declares that the research was conducted in the absence of any commercial or financial relationships that could be construed as a potential conflict of interest.
